# Effect of simvastatin on postoperative complications in patients undergoing one-lung ventilation during surgery: the Prevention HARP-2 randomised controlled trial

**DOI:** 10.1136/thorax-2025-223072

**Published:** 2025-07-08

**Authors:** Adam Glass, Cecilia M O’Kane, Akesh Dhrampal, Eustace Fontaine, James Ryan, Mahmoud Loubani, Sridhar Rathinam, Babu Naidu, Ingeborg D Welters, Jon Silversides, Bilal Alkhaffar, Jeremy Hayden, Ewen Griffiths, David Chan, Annmarie Doran, Sorcha Toase, Christina Campbell, Ashley Agus, Gavin D Perkins, Daniel Francis McAuley, Murali Shyamsundar

**Affiliations:** 1 Wellcome-Wolfson Institute of Experimental Medicine, Queen’s University Belfast, Belfast, UK; 2 Belfast Health and Social Care Trust, Belfast, UK; 3 Norfolk and Norwich University Hospital, Norwich, UK; 4 Manchester University NHS Foundation Trust, Manchester, UK; 5 South Tees NHS Foundation Trust, Middlesbrough, UK; 6 Castle Hill Hospital, Cottingham, UK; 7 Thoracic Surgery, Glenfield Hospital, Leicester, UK; 8 University of Birmingham, Birmingham, UK; 9 Critical Care, Royal Liverpool University Hospital, Liverpool, UK; 10 University of Manchester, Manchester, UK; 11 St James’s University Hospital, Leeds, UK; 12 University Hospitals Birmingham, Birmingham, UK; 13 University of Plymouth, Plymouth, UK; 14 Northern Ireland Clinical Trials Unit, Belfast, UK; 15 Health Economics, Northern Ireland Clinical Trials Unit, Belfast, UK; 16 Clinical Trials Unit, University of Warwick, Coventry, UK

**Keywords:** ARDS, Critical Care, Thoracic Surgery

## Abstract

**Rationale:**

Surgeries that require one-lung ventilation have high rates of postoperative cardiopulmonary complications with associated morbidity and mortality. Statins may limit inflammation involved in the development of these complications.

**Objectives:**

We tested the hypothesis that perioperative simvastatin use reduces postoperative cardiopulmonary complications, compared with placebo, in surgery requiring one-lung ventilation.

**Methods:**

Randomised, double-blind, multicentre trial of simvastatin versus placebo in patients undergoing elective oesophagectomy, lobectomy or pneumonectomy at 15 sites throughout the UK. Planned sample size is 452 patients. Participants were randomised to either simvastatin 80 mg or placebo for 4 days preoperatively and up to 7 days postoperatively.

**Measurements:**

The primary outcome measure was a composite endpoint of the incidence of acute respiratory distress syndrome, postoperative pulmonary complications, myocardial infarction and/or myocardial ischaemia during the first 7 days postoperatively or until hospital discharge. A modified intention-to-treat analysis excluded patients who did not receive the intervention preoperatively or proceed with the planned surgery.

**Main results:**

251 patients were randomised, 126 assigned to simvastatin and 125 to placebo, with 208 included in the modified intention-to-treat population. The trial was stopped early because of futility following recommendations from the data monitoring and ethics committee. The primary outcome occurred in 45/106 patients (42.5%) in the simvastatin group and 39/102 patients (38.2%) in the placebo group (OR 1.19 (95% CI 0.68 to 2.08); p=0.54). Secondary and safety outcomes were similar between the groups.

**Conclusion:**

In patients undergoing one-lung ventilation, simvastatin did not reduce the incidence of postoperative cardiopulmonary complications.

**Trial registration number:**

isrctn.org identifier, ISRCTN48095567.

**WHAT IS ALREADY KNOWN ON THIS TOPIC**

- Surgeries that require one-lung ventilation, such as oesophagectomy and lung resection, have high rates of postoperative cardiopulmonary complications. Previous perioperative studies, in surgeries not requiring one-lung ventilation, have shown that statins may limit the development of cardiopulmonary complications.

**WHAT THIS STUDY ADDS**

- In this randomised clinical trial of 251 patients undergoing one-lung ventilation for surgery the perioperative initiation of simvastatin did not reduce the incidence of postoperative cardiopulmonary complications.

**HOW THIS STUDY MIGHT AFFECT RESEARCH, PRACTICE OR POLICY**

- These findings do not support the perioperative initiation of simvastatin, in patients undergoing one-lung ventilation, to reduce the incidence of postoperative cardiopulmonary complications.

## Introduction

 Surgeries that require one-lung ventilation (OLV), such as oesophagectomy and lung resection, have high rates of postoperative cardiopulmonary complications. Postoperative pulmonary complications (PPCs) occur after 13–43% of such cases^1^,^3^ while myocardial infarction (MI) and myocardial injury after non-cardiac surgery (MINS) occur in 8–16% of cases.^4 5^ These complications have high morbidity and mortality rates, with increased intensive care unit admission rates and length of stay,^6^,^8^ and their occurrence in the first 30 days postoperatively is a strong determinant of both short-term and long-term mortality.^9^ Therapies which reduce the incidence of cardiopulmonary complications may therefore have the potential to improve long-term patient outcomes after major thoracic surgery.

Pulmonary inflammation secondary to OLV is a potential mechanism for the development of PPCs, and atherosclerotic plaque instability is a key mechanism for postoperative MI.^10^ Statins have significant immunomodulatory properties and may limit some of the underlying processes involved in the development of cardiopulmonary complications through hydroxy-methylglutaryl coenzyme A reductase inhibition. For example, simvastatin has been shown to reduce pulmonary inflammation and injury in humans in vivo models^11^ and in a single-centre trial in patients undergoing oesophagectomy.^12^ A meta-analysis of over 2000 patients undergoing cardiac, non-cardiac or vascular surgery revealed that preoperative statin use was associated with a significant reduction in postoperative MI, atrial fibrillation and length of hospital stay.^13^ Further studies have shown that statin use is associated with reduced PPCs and cardiovascular complications in patients undergoing cardiovascular surgery,^14^ non-cardiac surgery,^15^ pulmonary resection^16^ and oesophagectomy.^12^

The aim of this trial was to test the hypothesis that perioperative administration of simvastatin 80 mg daily reduces postoperative cardiopulmonary complications, when compared with placebo, in patients undergoing elective oesophagectomy, lobectomy or pneumonectomy surgery.

## Methods

### Trial design

The Prevention HARP-2 study was a randomised, double-blind, placebo-controlled multicentre trial of simvastatin in patients undergoing elective oesophagectomy, lobectomy or pneumonectomy. The study was conducted at 15 hospitals throughout the UK. The International Standard Randomised Controlled Trial Registry number for trial registration was ISRCTN48095567. The trial was sponsored by the Belfast Health and Social Care Trust and was coordinated by the Northern Ireland Clinical Trials Unit. All patients provided written informed consent. The study was conducted in accordance with the published study protocol.^17^ The study commenced with patients undergoing oesophagectomy with OLV, and the recruitment criteria were amended in 2017 to include patients undergoing lobectomy and pneumonectomy to increase the number of potential patients eligible for recruitment.

### Sites and participants

Adult patients (≥18 years) undergoing OLV for elective oesophagectomy, lobectomy or pneumonectomy were eligible. The main exclusion criteria were (1) age <18 years; (2) known active liver disease or abnormal liver function tests; (3) renal impairment; (4) inability to take medication enterally preoperatively; (5) use of feeding tube preoperatively; (6) current treatment with statins; (7) previous adverse reaction to statins; and (8) concomitant use of medications contraindicated with statin use. Further details of the eligibility criteria are included in the online supplemental text. If agreeable, written informed consent was obtained, following a face-to-face discussion about the study.

### Randomisation

Eligible participants were allocated preoperatively to either simvastatin 80 mg or placebo using an automated randomisation system in a 1:1 allocation ratio with a variable block size stratified by centre. The randomisation schedule was prepared before the start of the trial by the trial statistician. On randomisation, a unique medication pack identification number was allocated to the patient by the randomisation service, and the corresponding pack was dispensed to the patient by the blinded hospital pharmacy at the respective site. Patients, clinicians and the study team were blinded to each patient’s treatment allocation. Intervention and placebo drugs were identically packaged in white, opaque, high-density polyethylene plastic containers with a tamper-evident seal labelled with a unique medication pack identifier.

### Interventions

The trial drug was taken orally for 4 days preoperatively and either through a feeding tube or orally postoperatively for up to 7 days. In the event of surgery postponement, the patient stopped the study drug and restarted 4 days before the rescheduled surgery date. The total duration of the course was 11 days if there was no postponement of surgery and up to 14 days if surgery was postponed.

The study drug was discontinued if any one of the prespecified conditions was met prior to the maximum treatment period (up to 14 days from the start of the study drug); see online supplemental text.

The usual standard of care at sites was allowed. Interventions with anti-inflammatory effects, such as non-steroidal anti-inflammatory drugs and corticosteroids, were only used if deemed necessary and not contraindicated; their use was captured in the case report form.

Adherence to the study drug for self-administered doses was monitored via a patient diary. Patients were withdrawn from analysis if no study drug had been taken prior to surgery. All patients were included in the safety analysis.

### Outcome measures

The primary outcome measure was a composite endpoint of the incidence of acute respiratory distress syndrome (ARDS), PPC, MI or MINS^4^ during the first 7 days postoperatively or hospital discharge if earlier. These end points were chosen based on their effect on short-term and long-term outcomes and a biological rationale for simvastatin in modulating these endpoints. ARDS was defined according to the Berlin definition.^18^ PPC was defined by the Melbourne Group Scale as the occurrence of ≥4 of (1) temperature >38°C; (2) white cell count >11.2 or respiratory specific antibiotics; (3) physician diagnosis of pneumonia/chest infection; (4) chest X-ray report of atelectasis/consolidation; (5) production of purulent (yellow/green) sputum differing from preoperative status; (6) positive signs on sputum microbiology; (7) oxygen saturations <90% on room air or (8) readmission to or prolonged stay (over 36 hours) on the intensive care unit or high dependency unit for respiratory problems.^19^ MI was defined as the increase of at least one troponin value above the 99th percentile upper reference limit and one of the following criteria (1) symptoms of ischaemia; (2) new or presumed new significant ST segment or T wave ECG changes or new left bundle branch block; (3) development of pathological Q waves on ECG; radiological or echocardiographic evidence of new loss of viable myocardium or new regional wall motion abnormality; (4) identification of an intracoronary thrombus at angiography or autopsy.^20^ MINS was defined by a peak troponin T≥0.03 ng/mL in the first 3 days after surgery.^4^

Secondary outcomes were (1) mortality at days 28 and 90 following surgery and (2) ventilator-free days,^21^ defined as the number of days in the first 28 days following surgery that a patient was free from ventilator assistance for more than 48 hours. Within 28 days of surgery or hospital discharge if earlier (3) ARDS, PPC or MI; (4) atrial fibrillation; (5) venous thromboembolism; and (6) incidence and nature of any surgical complications. Within 7 days of surgery or hospital discharge if earlier (7) ARDS; (8) PPC; (9) MI; and (10) MINS.

Safety and health economic outcomes are detailed in the online supplemental text. The results of the health economic evaluation will be reported separately.

### Statistical analysis

Assuming an incidence rate of the composite endpoint of 25% by 7 days, a total sample size of 406 patients was needed to have 90% power at a two-tailed significance level of 0.05 to detect a 50% relative reduction in the incidence of the composite endpoint. The incidence rate of the composite outcome was based on a conservative estimate of the incidence of PPC, ARDS, MINS and MI individually, as there was no study that investigated the combined incidence of the individual components in a single cohort.^17^ Allowing for a 10% dropout rate gave an overall sample size of 452 (226 per arm).

A pre-planned modified intention-to-treat (MITT) analysis was performed including all randomised patients who proceeded with the planned surgery and had at least one preoperative dose of study drug, as stated in the statistical analysis plan. Due to the preoperative randomisation and starting the study intervention prior to surgery, a number of patients did not proceed with their planned surgery (eg, not proceeding to surgery due to a change to palliative care or change to planned surgery) or did not receive preoperative study drugs. The primary outcome was compared between treatment groups using a χ^2^ test.

A pre-planned secondary analysis was performed in which the primary outcome was the dependent variable and treatment allocation, centre, type of surgery and age were included as covariates in a logistic regression model. These covariates were selected due to the clinical importance and potential difference in the incidence of the primary outcome between surgical insults and patient age, as well as to account for any difference in centre-specific outcomes. An additional model included dexamethasone use as an additional covariate based on the potential anti-inflammatory effect of dexamethasone to influence the incidence of the primary outcome. A per-protocol analysis was performed for the primary outcome on patients who completed the treatment as allocated (ie, at least one preoperative dose of study drug and postoperative doses until day 7 or discharge) and proceeded with the planned surgery.

The prespecified subgroup analyses were chemotherapy prior to surgery (yes/no), type of surgery (oesophagectomy, lobectomy, pneumonectomy), surgical technique (minimally invasive/hybrid/open), duration of OLV (≤120 min and >120 min), smoking status, receipt of postoperative study drug (yes/no) and pericardial opening (yes/no).

The categorical outcomes were analysed using logistic regression models, with treatment allocation as an independent variable along with centre, type of surgery and age as covariates, and the results are reported as ORs with 95% CIs. Continuous outcomes were analysed using linear regression models, with treatment allocation as an independent variable and terms for centre, type of surgery and age in the models. Outcomes were reported as mean difference and 95% CIs. Again, both models were repeated with dexamethasone as a further covariate.

Baseline characteristics, follow-up measurements and safety data were described using appropriate descriptive summary measures depending on the scale of measurement and distribution. Missing data were not imputed.

A pre-planned interim analysis was conducted to analyse efficacy and safety parameters when approximately 50% of the planned number of patients had completed day 28 assessment or were discharged from hospital, whichever was sooner. There was no formal prespecified stopping criteria for futility. In relation to efficacy, a χ^2^ test was applied with a p value<0.001 according to the Haybittle-Peto stopping rule

### Role of the funding source

The trial was funded by a Health and Social Care Research and Development grant (Funder reference no. CDV/5137/15). The funder of the study had no role in study design, data collection, data analysis, data interpretation or writing of the report.

## Results

The study commenced recruitment on 28 October 2016. Recruitment was impacted by the COVID-19 pandemic, with recruitment temporarily paused at individual centres. Following the prespecified interim analysis of >50% of the intended sample size, the independent data monitoring and ethics committee recommended that the trial be stopped due to futility. There were no formal stopping rules for futility, and the decision to stop the study was based on the opinion of the data monitoring and ethics committee after considering all available information, including data from the interim analysis and feasibility of future recruitment, rather than a formal calculation of futility. The recommendation was accepted by the trial steering committee and agreed by the study sponsor and the trial was stopped on 10 September 2022.

**Figure 1:**
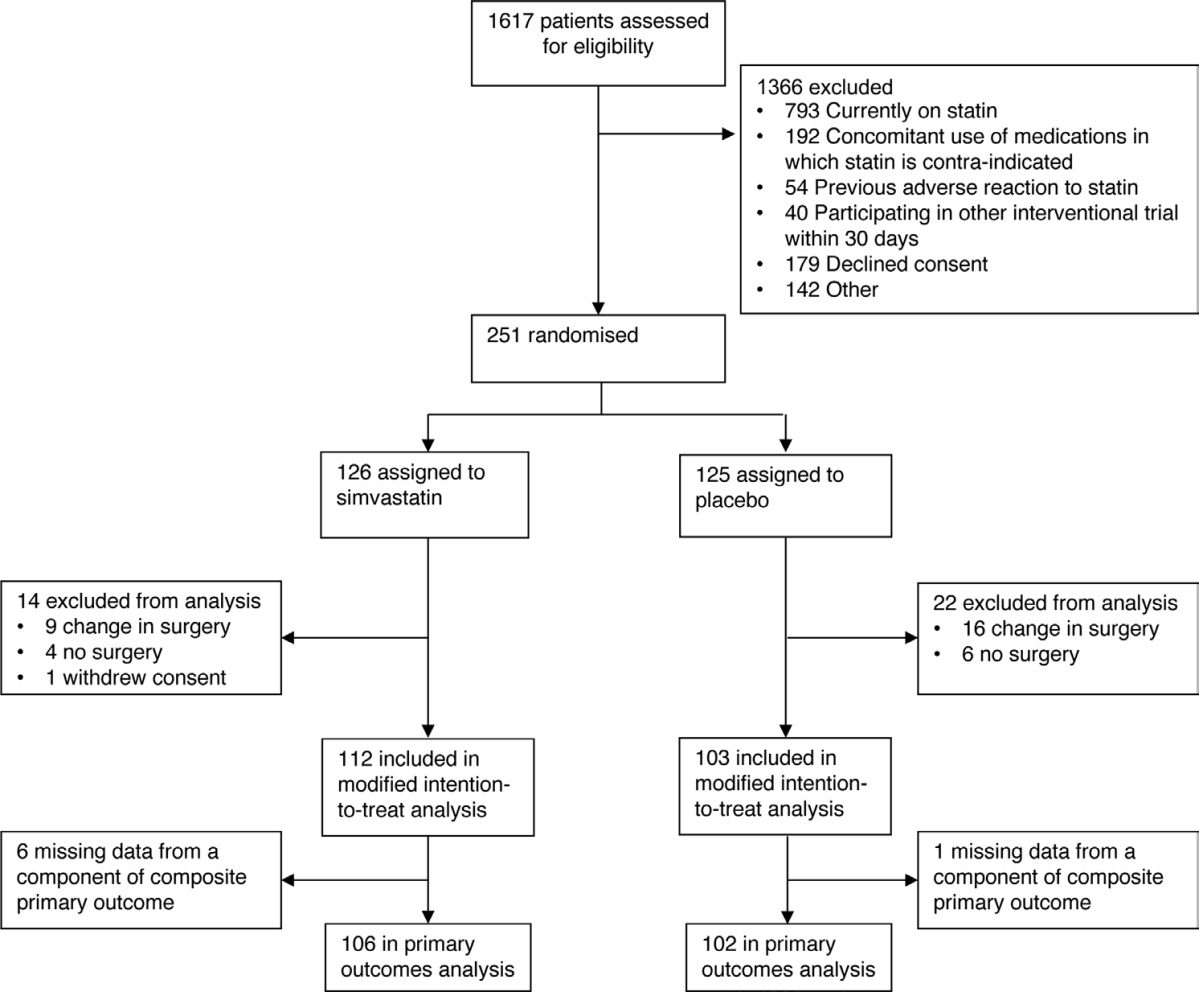
CONSORT (Consolidated Standards of Reporting Trials) diagram for screening, randomisation and follow-up of the study participants. Change in surgery, either following randomisation or intraoperatively resulting in the surgery performed no longer meeting inclusion criteria (eg, surgically inoperable or limited wedge resection of the lung). No surgery, procedure cancelled following randomisation (eg, patient management changed to palliative care). Of note, patients may meet more than one exclusion criterion.

In total, 1617 patients were screened for eligibility, 1366 of whom were excluded, with prior statin use the most common reason (793 patients), figure 1. A total of 251 patients were enrolled: 126 were randomly assigned to receive simvastatin and 125 to receive placebo. 35 patients were excluded following preoperative randomisation either because no surgery took place (eg, change to palliative care) or because a change in surgery performed so they no longer met inclusion criteria (eg, surgically inoperable or wedge resection of the lung). One patient withdrew consent for the use of any data following randomisation, resulting in 215 patients in the MITT analysis, with 112 patients in the simvastatin group and 103 patients in the placebo group. Complete composite primary outcome data were missing for six patients in the simvastatin group and one patient in the placebo group. These patients were excluded from primary outcome analysis only. Secondary outcomes are presented for the MITT population, and safety outcomes are presented for all patients enrolled in the trial except for the patient who withdrew consent.

Baseline demographics are presented for the MITT population in table 1. Characteristics were similar between groups. Patients received a mean of 3.6 (SD 0.8) and 3.6 (0.9) doses preoperatively and 5.5 (2.4) and 5.3 (2.2) doses postoperatively, with 68.8% and 68.0% of patients receiving the intervention for either 7 days postoperatively or until hospital discharge in the simvastatin and placebo groups respectively, table 2.

**Table 1:** Baseline demographic and clinical characteristics

	Simvastatin (n=112)	Placebo (n=103)
Age, mean (SD), years	62.9 (10.8)	63.3 (10.4)
Male sex, No. (%)	78 (69.6)	64 (62.1)
Height, mean (SD), cm	171.8 (10.0)	170.8 (10.3)
Weight, mean (SD), kg	78.2 (15.6)	79.7 (18.3)
Smoking status, No. (%)		
Current	14 (12.5)	16 (15.5)
Previous	66 (58.9)	58 (56.3)
Never	32 (28.6)	29 (28.2)
Past medical history, No. (%)		
Angina	0 (0.0)	0 (0.0)
Myocardial Infarction	1 (0.9)	1 (1.0)
Chronic obstructive pulmonary disease	13 (11.6)	11 (10.7)
Diabetes	4 (3.6)	1 (1.0)
Preoperative chemotherapy	57 (50.9)	48 (46.6)
If chemotherapy, no. of cycles	3.4 (1.0) n=57	3.4 (0.9) n=48
Cancer diagnosis, No. (%)		
Adenocarcinoma	75 (67.0)	56 (54.4)
Squamous cell	13 (11.6)	15 (14.6)
Barrett’s oesophagus	6 (5.4)	6 (5.8)
Small cell carcinoma	0 (0)	1 (1.0)
Other	18 (16.1)	25 (24.3)
Type of surgery, No. (%)		
Oesophagectomy	63 (56.3)	56 (54.4)
Lobectomy	44 (39.3)	43 (41.8)
Pneumonectomy	3 (2.7)	2 (1.9)
Other	2 (1.8)	2 (1.9)

Data are presented as the means (SDs) or n (%).

**Table 2:** Treatment after trial entry

	Simvastatin (n=112)	Placebo (n=103)
Study drug given preoperatively (at least one dose)	112 (100.0)	103 (100.0)
No. of doses preoperatively	3.6 (0.8)	3.6 (0.9)
Study drug given postoperatively (at least one dose)	111 (99.1)	100 (97.1)
Study drug given postoperatively (all doses)	66 (59.5)	60 (60.0)
No. of doses postoperatively	5.5 (2.4)	5.3 (2.2)
Reasons for termination of study drug		
7 days after surgery	42 (37.5)	38 (36.9)
Discharge from hospital	35 (31.3)	32 (31.1)
Study drug-related adverse event	7 (6.3)	11 (10.7)
Development of clinical condition requiring immediate treatment with a statin or other drugs which interact with statins	8 (7.1)	6 (5.8)
Discontinuation of active medical treatment	0 (0.0)	0 (0.0)
Patient’s request for withdrawal from the study	0 (0.0)	0 (0.0)
Decision by the attending clinician that study drug should be discontinued on safety grounds	4 (3.6)	1 (1.0)
Death	0 (0.0)	0 (0.0)
Change of surgery	0 (0.0)	0 (0.0)
Patient nil by mouth	9 (8.0)	9 (8.7)
Other	7 (6.3)	6 (5.8)

Data are presented as the means (SDs) or n (%).

There was no difference in the incidence of the composite primary outcome between groups, occurring in 45/106 patients (42.5%) in the simvastatin group and 39/102 patients (38.2%) in the control group (OR 1.19 (95% CI 0.68 to 2.08); p=0.54, table 3. Similarly, the per-protocol analysis did not reveal a difference in outcome between groups, occurring in 19/61 patients (31.2%) in the simvastatin group and 17/59 patients (28.8%) in the placebo group (OR 1.12 (95% CI 0.51 to 2.44); p=0.78).

**Table 3:** Primary and secondary outcome measures

	Simvastatin	Placebo	OR (95% CI)	P value
Primary outcome*	n=106	n=102		
Incidence of ARDS, PPC, MI or MINS to day 7	45 (42.5)	39 (38.2)	1.19 (0.68 to 2.08)	0.536
Secondary outcomes†	n=112	n=103		
28-day mortality	3 (2.7)	2 (2.0)	1.40 (0.22 to 8.79)	0.720
90-day mortality	4 (3.6)	3 (2.9)	1.15 (0.24 to 5.55)	0.893
Ventilator-free days‡	26.5 (5.9)	26.9 (4.8)	−0.35 (−1.83 to 1.13)	0.640
ARDS within 28 days of surgery or hospital discharge if earlier	9 (8.2)	6 (5.9)	1.29 (0.43 to 3.86)	0.654
PPC within 28 days of surgery or hospital discharge if earlier	31 (28.2)	25 (24.3)	1.14 (0.61 to 2.14)	0.677
MI within 28 days of surgery or hospital discharge if earlier	4 (3.8)	1 (1.0)	3.25 (0.35 to 30.46)	0.302
AF within 28 days of surgery or hospital discharge if earlier	20 (18.0)	24 (23.5)	0.66 (0.33 to 1.32)	0.236
VTE within 28 days of surgery or hospital discharge if earlier	3 (2.7)	1 (1.0)	2.94 (0.29 to 29.44)	0.360
Surgical complications within 28 days of surgery or hospital discharge if earlier	39 (34.8)	41 (39.8)	0.76 (0.43 to 1.34)	0.343
ARDS within 7 days of surgery or hospital discharge if earlier	7 (6.4)	5 (4.9)	1.26 (0.38 to 4.17)	0.701
PPC within 7 days of surgery or hospital discharge if earlier	25 (22.7)	24 (23.3)	0.91 (0.48 to 1.75)	0.785
MI within 7 days of surgery or hospital discharge if earlier	4 (3.7)	1 (1.0)	3.22 (0.34 to 30.19)	0.306
MINS within 7 days of surgery or hospital discharge if earlier	25 (23.6)	15 (15.0)	1.74 (0.84 to 3.62)	0.139

Data are presented as n (%).

* Result from χ2 test.

† Results from logistic regression model with the treatment group as an independent variable along with centre, type of surgery and age as a covariate.

‡ Point difference.

AF, atrial fibrillation; ARDS, acute respiratory distress syndrome; MI, myocardial infarction; MINS, myocardial injury after non-cardiac surgery; PPC, postoperative pulmonary complications; VTE, venous thromboembolism.

**Figure 2:**
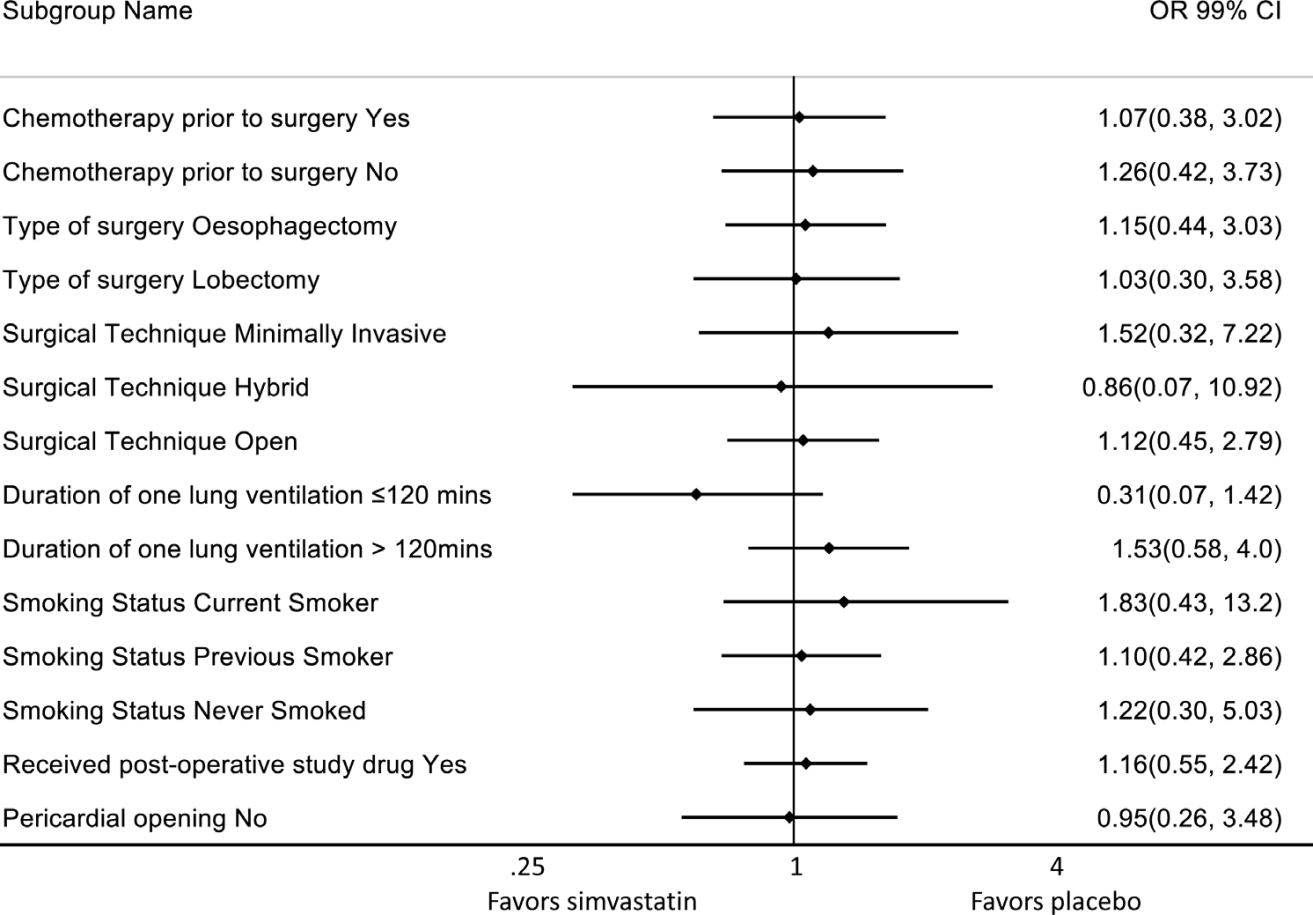
Prespecified subgroup analysis. ORs presented on a log scale.

There was no difference in the incidence of the primary outcome in the prespecified subgroups, figure 2.

Secondary outcomes were similar between the groups, table 3.

Simvastatin was well tolerated, with safety outcomes similar between the groups. One patient (in the simvastatin group) developed postoperative AKI by day 7. Three serious adverse events occurred in the study, none were deemed related to the study drug. The rates of adverse events were similar between the groups, table 4.

**Table 4:** Safety outcomes

	Simvastatin (n=125)	Placebo (n=125)	Risk ratio (95% CI)	P value
Blood tests				
CK>10 times ULN (n, %)	6 (4.8)	8 (6.4)	0.75 (0.27 to 2.10)	0.784
ALT>5 times ULN (n, %)	9 (7.2)	5 (4.0)	1.80 (0.62 to 5.22)	0.410
AST>5 times ULN (n, %)	3 (2.4)	1 (0.8)	3.00 (0.32 to 28.45)	0.622
Acute kidney injury (n, %)	1 (1.4)	0 (0.0)		
Adverse events				
Total AEs – no.	32	25	1.15 (0.67 to 1.98)	0.738
AEs related to study drug – no.	20	16	1.29 (0.67 to 2.47)	0.571
Total SAEs – no.	1	2	0.5 (0.05 to 5.44)	1.00
SAEs related to study drug – no.	0	0		
SAEs related to study drug and unexpected – no.	0	0		

Data are presented as n (%). The χ2 test results are presented alongside the risk ratio and 95% CI.

AE, adverse event; ALT, alanine aminotransferase; AST, aspartate aminotransferase; CK, creatine kinase; SAE, serious adverse event; ULN, Upper limit of normal.

## Discussion

This multicentre randomised control trial aimed to investigate whether perioperative simvastatin therapy would reduce cardiopulmonary complications in patients undergoing OLV for oesophagectomy, lobectomy or pneumonectomy. The study did not find any difference in the composite primary outcome of cardiopulmonary complications or in any of the secondary outcomes between simvastatin and placebo groups and therefore does not support the perioperative initiation of simvastatin therapy for prevention of cardiopulmonary complications in patients undergoing OLV for oesophagectomy, lobectomy or pneumonectomy.

Previous surgical studies have tested a variety of statins (atorvastatin, fluvastatin, rosuvastatin, pravastatin and simvastatin) and durations of treatment (one single additional dose to months of treatment) in both statin-naïve patients and patients established on statin therapy. These studies have predominantly investigated cardiovascular complications, with heterogeneous results across studies and surgical specialties. In the most comparable study to this one in terms of population, Berwanger and colleagues initiated atorvastatin or placebo in 648 statin-naïve patients with cardiovascular risk factors who underwent non-cardiac surgery (6% vascular). There was no difference in the composite outcome of death, myocardial injury and/or stroke within 30 days or in any secondary outcome,^22^ similar to our findings. While several large trials of statin therapy have been undertaken in established ARDS, such as SAILS^23^ and HARP-2,^24^ finding no clinical benefit overall, few perioperative studies have investigated the impact of perioperative statin initiation on the incidence of pulmonary injury or postoperative pulmonary complications.

Of note, an interaction between OLV duration and simvastatin effect was observed. OLV duration ≤120 min showed a trend to benefit from simvastatin but to harm when OLV>120 min, online supplemental eTable 1. This may be driven by the higher primary outcome incidence in the OLV≤120 min placebo group, 15/22 patients (68.2%), however, the low number of patients in this comparison limits interpretation.

The lack of benefit of simvastatin in this study could be due to the selection of the wrong statin, suboptimal dosing, too short a duration of therapy or the incorrect population being tested. Alternatively, the original hypothesis may have been incorrect.

Simvastatin 80 mg was selected following an in vivo study demonstrating reduced pulmonary and systemic inflammation in a human model of lung injury^11^ and a subsequent pilot study from our group.^12^ The 80 mg dose has subsequently been shown to be beneficial in patients with COVID-19^25^ and in a secondary analysis of an ARDS study.^26^ The preoperative duration of intervention was based on our human model study, which demonstrated amelioration of the inflammatory response to inhaled endotoxin in healthy volunteers pretreated with simvastatin for 4 days^11^ and the postoperative duration was selected because the majority of postoperative complications occurred within the first 7 days postoperatively.^27^ There is a suggested benefit of prolonged statin use preoperatively. Schouten and colleagues demonstrated a reduction in myocardial ischaemia following vascular surgery in a randomised controlled trial of 497 patients with a median duration of 37 days preoperative fluvastatin administration.^10^

The study population was selected due to the requirement for OLV and high rates of cardiopulmonary complications. The incidence of the composite endpoint in our study was greater than anticipated based on the pilot study,^17^ but was comparable to recently published literature^28^,^30^ with a higher incidence of MINS than a general surgical population.^31^ Of note, there may be an additional mechanism of myocardial injury with OLV related to the acute intraoperative increase in right ventricular afterload during OLV, which may cause a local inflammatory response in the right ventricle.^32^ The effect of simvastatin on this mechanism is unknown. In spite of this, the perioperative inflammatory response is likely to be of significantly lesser magnitude than that observed in patients with established ARDS, potentially resulting in a reduced anti-inflammatory effect from simvastatin.

Secondary analysis of the SAILS^23^ and HARP-2^24^ studies revealed two underlying ‘hypoinflammatory’ and ‘hyperinflammatory’ phenotypes,^26 33^ and in the HARP-2 trial, simvastatin therapy was suggested to be beneficial in patients with a hyperinflammatory phenotype.^24 26^ In the simvastatin arm of the REMAP-CAP platform trial, there was a 95.9% posterior probability of efficacy, suggesting a benefit of simvastatin therapy.^25^ There was a suggestion that this benefit was greater in patients with elevated C-reactive protein and ferritin, although these are poor discriminators of the hyperinflammatory phenotype. If simvastatin does have beneficial anti-inflammatory effects, the benefit may only be in patients with evidence of the highest levels of systemic inflammation. Cluster analysis of a perioperative cohort has suggested a potentially analogous perioperative phenotype characterised by higher inflammation with a higher rate of postoperative pulmonary complications.^34^

In addition, the effect of simvastatin may be attenuated by immunosuppression from chemotherapy and/or the anti-inflammatory effect of perioperative steroid administration as an antiemetic agent. However, there was no interaction between prior chemotherapy and treatment with simvastatin. Furthermore, the inclusion of dexamethasone administration as a covariate did not influence the results of any of the primary or secondary outcomes, online supplemental eTable 2.

This study has several limitations. Only approximately 16% of the screened patients were included in the study, which may limit the generalisability of the results. Based on previous data,^12 24^ we anticipated that 30% of patients would be ineligible due to pre-existing statin use; however, the rate in this study was almost 50%. Furthermore, 246/1617 (15%) patients screened had either a contraindication to statin use or a previous adverse reaction to a statin; therefore, the recruitment rate is likely due to the specific intervention used in the trial. In addition, the number of patients excluded after randomisation could have introduced a degree of bias, although given the reasons patients were excluded, this risk is likely to be low.^35^ While the premature closure of the trial may have increased the risk of incorrectly rejecting the null hypothesis, there was no trend of benefit of treatment in the primary or secondary outcomes. The study was powered to detect a 50% relative risk reduction. While this is an ambitious target for a single intervention, this was based on the results of our pilot trial^12^ and the study of Schouten and colleagues^10^ in vascular surgery. The individual components of the composite primary outcome differ in both incidence and severity, although there is no difference between the groups in any of the individual components of the composite primary outcome, as detailed in the secondary outcomes. While a lesser relative risk reduction may still have resulted in a clinically important difference, there was no evidence that there was any trend to a reduction in risk of the primary or secondary outcomes. The decision to extend the recruitment population to include patients undergoing anatomical lung resection as well as patients undergoing oesophagectomy may have recruited a heterogeneous population at different risk of postoperative complications. This may have been further confounded by variation in preoperative and perioperative risk factors for postoperative complications. However, as the groups were well matched would suggest this was not a major factor to account for our findings. Finally, while clinical teams were advised to adopt a standardised management strategy, we did not strictly protocolise anaesthetic or surgical care to maintain the generalisability of the results, which may have introduced a degree of variation in patient care.

In conclusion, perioperative simvastatin administration does not reduce cardiopulmonary complications compared with placebo following lung resection or oesophagectomy. These results do not support the perioperative initiation of simvastatin for this purpose.

## Supplementary material

10.1136/thorax-2025-223072 online supplemental file 1

10.1136/thorax-2025-223072 online supplemental file 2

10.1136/thorax-2025-223072 online supplemental file 3

## Data Availability

Data are available upon reasonable request.
