# Supplementary material for: Effect of simvastatin on postoperative complications in patients undergoing one-lung ventilation during surgery: the Prevention HARP-2 randomised controlled trial
Source: Thorax. 2025 Jul 8;81(4):e223072. doi: 10.1136/thorax-2025-223072 (PMC13018756; doi:10.1136/thorax-2025-223072)
Supplement: online supplemental file 1 [file thorax-81-4-s001.docx]

Supplementary tables.

eTable 1 Prespecified subgroup interaction analysis

|  | Simvastatin | Placebo | Odds Ratio (99% CI) | Interaction term (p) |
| --- | --- | --- | --- | --- |
| Chemotherapy prior to surgery |  |  |  |  |
| Yes | 28 (52.8%)  n=53 | 24 (51.1%)  n=47 | 1.07 (0.38 - 3.02) | 0.820 |
| No | 17 (32.1%)  n=53 | 15 (27.3%)  n=55 | 1.26 (0.42 - 3.73) |  |
| Type of surgery |  |  |  |  |
| Oesophagectomy | 30 (50.9%)  n=59 | 26 (47.3%)  n=55 | 1.15 (0.44 - 3.03) | 0.830 |
| Lobectomy | 12 (28.6%)  n=42 | 12 (27.9%)  n=43 | 1.03 (0.30 - 3.58) |  |
| Pneumonectomy | 3 (100.0%)  n=3 | 1 (50.0%)  n=2 | - |  |
| Other | 0 (0.0%)  n=2 | 0 (0.0%)  n=2 | - |  |
| Surgical Technique |  |  |  |  |
| Minimally Invasive | 8 (30.8%)  n=26 | 7 (22.6%)  n=31 | 1.52 (0.32 - 7.22) | 0.884 |
| Hybrid | 3 (50.0%)  n=6 | 7 (53.9%)  n=13 | 0.86 (0.07 - 10.92) |  |
| Open | 34 (46.0%)  n=74 | 25 (43.1%)  n=58 | 1.12 (0.45 - 2.79) |  |
| Duration of one lung ventilation |  |  |  |  |
| ≤ 120 mins | 12 (40.0%)  n=30 | 15 (68.2%)  n=22 | 0.31 (0.07 - 1.42) | 0.017 |
| > 120mins | 26 (43.3%)  n=60 | 21 (33.3%)  n=63 | 1.53 (0.58 - 4.0) |  |
| Smoking Status |  |  |  |  |
| Current Smoker | 8 (61.5%)  n=13 | 7 (46.7%)  n=15 | 1.83 (0.43 - 13.2) | 0.831 |
| Previous Smoker | 26 (41.9%)  n=62 | 23 (39.7%)  n=58 | 1.10 (0.42 - 2.86) |  |
| Never Smoked | 11 (35.5%)  n=31 | 9 (31.0%)  n=29 | 1.22 (0.30 - 5.03) |  |
| Received postoperative study drug |  |  |  |  |
| Yes | 44 (41.9%)  n=105 | 38 (38.4%)  n=99 | 1.16 (0.55 - 2.42) | - |
| No | 1 (100.0%)  n=1 | 1 (33.3%)  n=3 | - |  |
| Pericardial opening |  |  |  |  |
| Yes | 1 (50.0%)  n=2 | 0 (0.0%)  n=1 | - | - |
| No | 11 (30.6%)  n=36 | 12 (31.6%)  n=38 | 0.95 (0.26 - 3.48) |  |

Data are presented as n (%). Odds ratios and 99% CI from the treatment x subgroup interaction models.

eTable 2 Logistic regression including dexamethasone

|  | **Simvastatin** | **Placebo** | **Odd ratio (95% CI); p value** | **P value** |
| --- | --- | --- | --- | --- |
| Primary outcome | n = 106 | n =102 |  |  |
| Incidence of ARDS, PPC, MI or MINS to day 7 | 45 (42.5%) | 39 (38.2%) | 1.2 (0.6 - 2.3) | 0.548 |
|  |  |  |  |  |
| Secondary outcomes | n = 112 | n= 103 |  |  |
| 28 day mortality | 3 (2.7%) | 2 (2.0%) | 1.40 (0.22 - 8.79) | 0.721 |
| 90 day mortality | 4 (3.6%) | 3 (2.9%) | 1.15(0.24 - 5.56) | 0.892 |
| Ventilator free days (adjusted) ^a^ | 26.4(0.5) | 27.1(0.6) | -0.72(-2.3 - 0.81) | 0.354 |
| ARDS within 28 days of surgery or hospital discharge if earlier | 9 (8.2%) | 6 (5.9%) | 1.27 (0.42 - 3.84) | 0.667 |
| PPC within 28 days of surgery or hospital discharge if earlier | 31 (28.2%) | 25 (24.3%) | 1.13 (0.60 - 2.13) | 0.717 |
| MI within 28 days of surgery or hospital discharge if earlier | 4 (3.8%) | 1 (1.0%) | 3.20 (0.34 - 30.22) | 0.309 |
| AF within 28 days of surgery or hospital discharge if earlier | 20 (18.0%) | 24 (23.5%) | 0.59 (0.29 - 1.21) | 0.154 |
| VTE within 28 days of surgery or hospital discharge if earlier | 3 (2.7%) | 1 (1.0%) | 2.91 (0.29 - 29.23) | 0.363 |
| Surgical complications within 28 days of surgery or hospital discharge if earlier | 39 (34.8%) | 41 (39.8%) | 0.73 (0.41 - 1.31) | 0.294 |
| ARDS within 7 days of surgery or hospital discharge if earlier | 7 (6.4%) | 5 (4.9%) | 1.26 (0.38 - 4.18) | 0.698 |
| PPC within 7 days of surgery or hospital discharge if earlier | 25 (22.7%) | 24 (23.3%) | 0.90 (0.47 - 1.73) | 0.745 |
| MI within 7 days of surgery or hospital discharge if earlier | 4 (3.7%) | 1 (1.0%) | 3.17 (0.34 – 29.98) | 0.314 |
| MINS within 7 days of surgery or hospital discharge if earlier | 25 (23.6%) | 15 (15.0%) | 1.68 (0.80 - 3.51) | 0.168 |

Data are presented as n (%). Logistic regression model with treatment group as an independent variable along with centre, type of surgery, age and dexamethasone use as a covariate. ^a^ point difference. ARDS = acute respiratory distress syndrome, PPC = post-operative pulmonary complications, MI = myocardial infarction, MINS = myocardial injury after non-cardiac surgery, AF = atrial fibrillation, VTE = venous thromboembolism.
