# Supplementary material for: Effect of simvastatin on postoperative complications in patients undergoing one-lung ventilation during surgery: the Prevention HARP-2 randomised controlled trial
Source: Thorax. 2025 Jul 8;81(4):e223072. doi: 10.1136/thorax-2025-223072 (PMC13018756; doi:10.1136/thorax-2025-223072)
Supplement: online supplemental file 2 [file thorax-81-4-s002.docx]

Supplementary text

**Detailed exclusion criteria**; 1) age <18 years, 2) known active liver disease (Child’s Pugh score >11), or abnormal liver function tests: alanine aminotransferase (ALT) / aspartate aminotransferase (AST) >3 times upper limit normal range (ULN) of local laboratory range, 3) renal impairment (calculated creatinine clearance less than 30mL/minute), 4) inability to take medication enterally pre-operatively, 5) use of feeding tube (nasogastric, gastric, jejunal) pre-operatively, 6) subject-reported lactose intolerance, 7) participation in other intervention trials within 30 days, 8) current treatment with statins, 9) known hypersensitivity to the study medication, 10) previous adverse reaction to statins, 11) concomitant use of fibrates or other lipid-lowering therapy, 12) concomitant use of itraconazole, ketoconazole, posaconazole, voriconazole, erythromycin, clarithromycin, telithromycin, human immunodeficiency virus protease inhibitors, boceprivir, telaprevir, nefazodone, cobicistat, cyclosporine, danazol, amiodarone, amlodipine, verapamil or diltiazem, fusidic acid, and niacin, 13) lack of informed consent indicating that they understand all the pertinent aspects of the trial prior to enrolment, and 14) currently pregnant or lactating

The exclusion criteria, “5) use of feeding tube (nasogastric, gastric, jejunal) pre-operatively” was added due to a change in the method of blinding from a matched placebo to over-encapsulation of simvastatin and placebo during the study. Opening of the capsule would therefore have led to unblinding of the participant.

**Study drug discontinuation conditions**; 1) study-drug-related adverse event, i.e. (a) creatinine kinase (CK) > 10 times the ULN of the local laboratory range, or (b) ALT/AST > 5 times the ULN of the local laboratory range, 2) development of a clinical condition requiring immediate treatment with a statin or other drugs which interact with statins, 3) discontinuation of active medical treatment, 4) patient’s request for withdrawal from the study, 5) decision by the attending clinician that the study drug should be discontinued on safety grounds, 6) discharge from hospital, 7) change of type of surgery or 8) death.

**Safety outcomes**; 1) CK >10 times the ULN (day 0, day 3, and day 7 post-operative) of the local laboratory range, 2) ALT or AST >5 times the ULN (day 0, day 3, and day 7) of the local laboratory range, 3) acute kidney injury defined according to Kidney Disease Improving Global Outcomes guidelines (using change from baseline serum creatinine) within 7 days of surgery, 4) serious adverse events (SAE), adverse events (AE), and occurrence of suspected unexpected serious adverse reactions (SUSARs).

**Health economic outcomes**; 1) health-related quality of life (baseline, day 90) measured using the EQ-5D-5L,^21^ 2) health service use up to 90 days.
