# Supplementary material for: Effect of simvastatin on postoperative complications in patients undergoing one-lung ventilation during surgery: the Prevention HARP-2 randomised controlled trial
Source: Thorax. 2025 Jul 8;81(4):e223072. doi: 10.1136/thorax-2025-223072 (PMC13018756; doi:10.1136/thorax-2025-223072)
Supplement: online supplemental file 3 [file thorax-81-4-s003.pptx]

## Slide 1
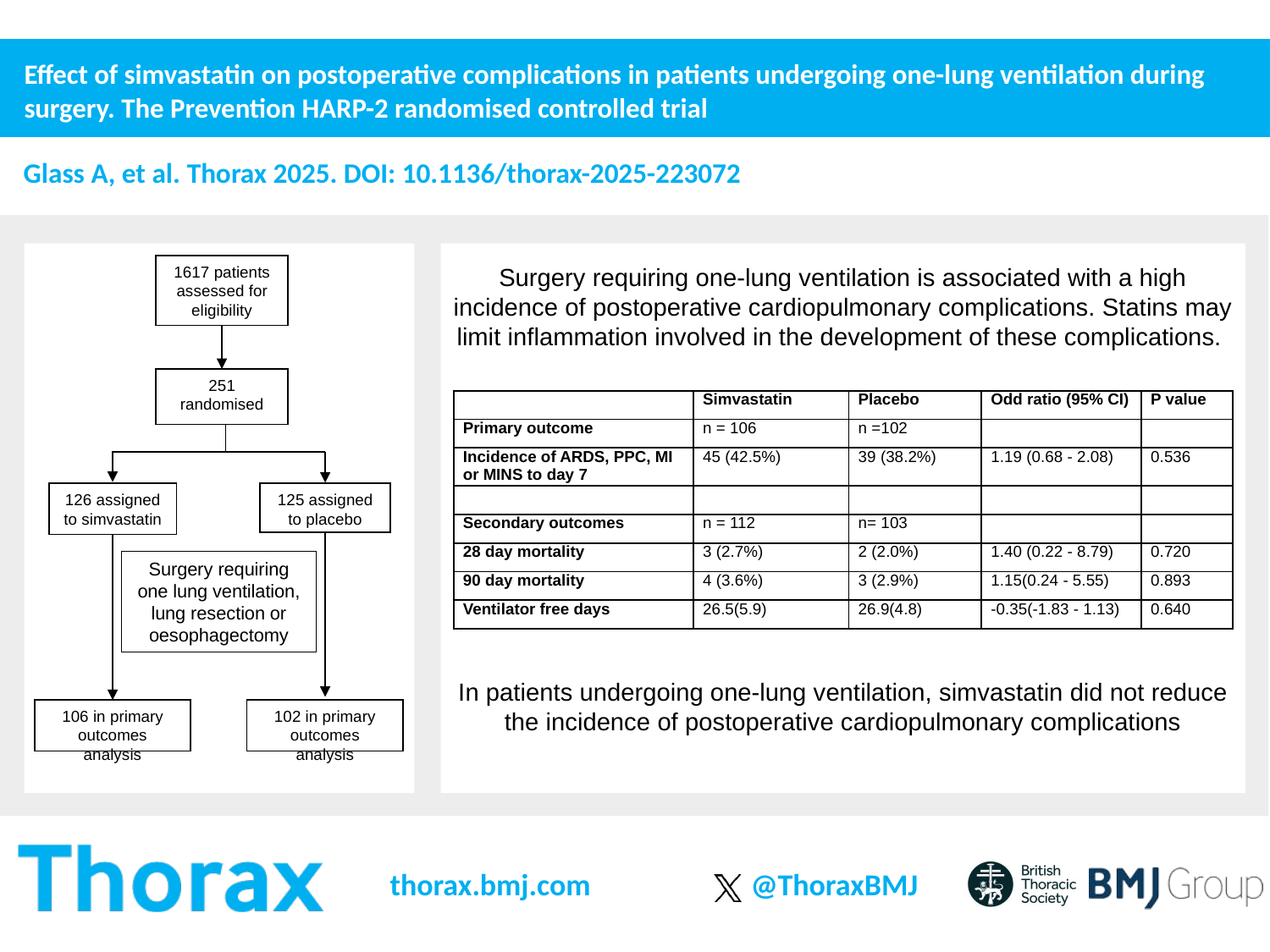

Effect of simvastatin on postoperative complications in patients undergoing one-lung ventilation during surgery. The Prevention HARP-2 randomised controlled trial
Glass A, et al. Thorax 2025. DOI: 10.1136/thorax-2025-223072
1617 patients assessed for eligibility
Surgery requiring one-lung ventilation is associated with a high incidence of postoperative cardiopulmonary complications. Statins may limit inflammation involved in the development of these complications.
Manuscript Title
251 randomised
| | Simvastatin | Placebo | Odd ratio (95% CI) | P value |
| --- | --- | --- | --- | --- |
| Primary outcome | n = 106 | n =102 | | |
| Incidence of ARDS, PPC, MI or MINS to day 7 | 45 (42.5%) | 39 (38.2%) | 1.19 (0.68 - 2.08) | 0.536 |
| | | | | |
| Secondary outcomes | n = 112 | n= 103 | | |
| 28 day mortality | 3 (2.7%) | 2 (2.0%) | 1.40 (0.22 - 8.79) | 0.720 |
| 90 day mortality | 4 (3.6%) | 3 (2.9%) | 1.15(0.24 - 5.55) | 0.893 |
| Ventilator free days | 26.5(5.9) | 26.9(4.8) | -0.35(-1.83 - 1.13) | 0.640 |
126 assigned to simvastatin
125 assigned to placebo
Surgery requiring one lung ventilation, lung resection or oesophagectomy
In patients undergoing one-lung ventilation, simvastatin did not reduce the incidence of postoperative cardiopulmonary complications
106 in primary outcomes analysis
102 in primary outcomes analysis
© Author(s) (or their employer(s) 2019. Re-use permitted under CC BY. Published by BMJ.
thorax.bmj.com @ThoraxBMJ
